# Antioxidant enzymes of *Pseudochlorella pringsheimii* under two stressors: variation of SOD Isoforms activity

**DOI:** 10.1007/s10265-023-01473-5

**Published:** 2023-06-13

**Authors:** Mostafa M. S. Ismaiel, Michele D. Piercey-Normore

**Affiliations:** 1 Botany and Microbiology Department, Faculty of Science, Zagazig University, Zagazig, 44519 Egypt; 2 Faculty of Science, Algoma University, Sault Ste Marie, Ontario P6A 2G4 Canada

**Keywords:** Antioxidant enzymes, Chlorophyta, Expression, Gel activity, SOD isoforms, FeSOD

## Abstract

Algae are always facing the challenge of exposure to different stress conditions, therefore raising challenges of adaptation for survival. In this context, the growth and the antioxidant enzymes of the green stress-tolerant alga *Pseudochlorella pringsheimii* were investigated under two environmental stresses viz. iron and salinity. The number of algal cells was moderately increased by iron treatment in the range of 0.025–0.09 mM of iron, yet, the number of cells decreased at high iron concentrations (0.18 to 0.7 mM Fe). Furthermore, the different NaCl concentrations (8.5–136.0 mM) had an inhibitory effect on the algal cell number, compared to the control.

The superoxide dismutase (SOD) showed three isoforms namely; Mn, Fe, and Cu/Zn SOD. The *in gel* and in vitro (tube-test) activities of FeSOD were higher compared with the other SOD isoforms. The activity of total SOD and its isoforms increased significantly by the different concentrations of Fe and non-significantly by NaCl. The maximum SOD activity was recorded at 0.7 mM Fe (67.9% above control). The relative expression of *FeSOD* was high under iron and NaCl at 8.5 and 34 mM, respectively. However, *FeSOD* expression was reduced at the highest NaCl tested concentration (136 mM). In addition, the antioxidant enzyme activity of catalase (CAT) and peroxidase (POD) were accelerated by increasing iron and salinity stress which indicates the essential role of these enzymes under stress. The correlation between the investigated parameters was also investigated. A highly significant positive correlation between the activity of total SOD and its isoforms, and with the relative expression of *FeSOD* was observed.

**Supplementary Information:**

The online version contains supplementary material available at 10.1007/s10265-023-01473-5.

## Introduction

Reactive oxygen species (ROS) are highly interactive oxygen-derivative compounds that include superoxide, hydroxyl radical, peroxides, singlet oxygen, and alpha-oxygen. They are normal byproducts found in the cells of living organisms e.g. bacteria, fungi, algae, and plants. ROS are regularly produced as a result of cellular metabolism including respiration and photosynthesis. They help in signaling and connection between the cellular organelles as mitochondria and chloroplasts, regulation of gene expression and hence the control of the cell cycle and growth (Cheng et al. 2016; Chien and Lin 2019; Luis et al. 2006; Nowicka 2022; Ugya et al. 2020; Wang and Ki 2020).

However, when exposed to environmental stressors (e.g. UV irradiation, high light intensity, high or low temperature, salinity, toxic pollutants as heavy metals, etc.) the concentration of ROS critically increases and all the cellular components must cope with excess ROS to diminish the harmful effect on bioactive components (Chen et al. 2015; Cheng et al. 2016; Chien and Lin 2019; Wang and Ki 2020; Yang et al. 2022). ROS may disrupt cellular homeostasis, making a state of oxidation or oxidative stress imposed on the living cells. Mutagenesis, metabolic disruption, and changes in cell structure can result from oxidative damage to DNA, proteins, and lipids (Okamoto et al. 2001; Ugya et al. 2020).

Environmental stress can also be caused by high iron concentration in the growth media of microorganisms. Iron density is 7.874 g cm^−^³ therefore it is considered a heavy metal (Nowicka 2022). It is essential for the growth of all living organisms as it is a component of the electron-transport system (generation of energy), biosynthesis of chlorophylls, carotenoids, and DNA, nitrogen assimilation, biosynthesis of fatty acids, and as a co-factor in several enzymes (El-Sheekh et al. 2022; Ismaiel et al. 2018; Nowicka 2022; Rybak et al. 2020; Yang et al. 2022). Iron is usually present in low concentration due to its partial solubilization, oxidation, precipitation, and complexation (with other salts), therefore it is considered a critical microelement for algal growth (Rybak et al. 2020; Torres et al. 2008; Yang et al. 2022). However, higher concentrations of iron may be recorded naturally due to the action of chemical or biological processes in water and soil, or due to increased anthropogenic activities (such as mining, industrial processes, etc.) which increase the heavy metal ions and pose a severe hazard to water and land ecosystems (Nowicka 2022). ROS production was highly promoted under high iron concentrations due to stimulation of the autooxidation process. For example, the production of hydroxide (OH^−^) and hydroxyl radicals (OHֺ) occurs when hydrogen peroxide (H_2_O_2_) reacts with Fe^2+^ (Fenton reaction). Moreover, the production of hydroxyl and hydroxide ions occurs when iron catalyzes the reaction between H_2_O_2_ and superoxide ion O_2_ֺ^−^ (Haber–Weiss cycle). Usually, mitochondria and chloroplasts produce the majority of ROS in living cells (Nowicka 2022; Yang et al. 2022).

Salinity, another example of an abiotic stress, is a state of high salt concentration (Na^+^, K^+^, Ca^2+^, Mg^2+^, and Cl^−^) in water and soil. High salinity may occur either naturally due to high temperature, drought, decay of soil, rocks, minerals by weather factors, etc., or artificially due to the irrigation, construction of roads, industry, etc. (Pietryczuk et al. 2014; Singh et al. 2018). The hazardous effect of high salinity may come from direct osmotic stress, imbalance of cellular ions, dehydration, and the high production of different ROS, which causes oxidative damage to the cell (Chen et al. 2015; Mallik et al. 2011; Pietryczuk et al. 2014; Singh et al. 2018; Wang and Ki 2020; Zhao et al. 2022).

Living organisms have developed different antioxidant mechanisms to lower the concentration of ROS and to survive stressful conditions. The role of antioxidant enzymes is vital for survival under stressful conditions because they reduce the harmful effects of ROS. By adjusting antioxidant enzymes, many attempts have been undertaken to increase the salt tolerance of plants (Hu et al. 2012; Jebara et al. 2005; Mallik et al. 2011; Mao et al. 2020). The induction of the antioxidant enzyme activities of Cyanophyta and Chlorophyta under several environmental conditions has been reported in the literature (Ajitha et al. 2019; Ismaiel and Said 2018; Önem et al. 2018; Ugya et al. 2020).

Superoxide dismutase (SOD) is an important antioxidant enzyme that, regarding its function, is considered the first and essential guard against ROS production. SOD dismutates the superoxide anions that are increased under stress conditions and convert them to hydrogen peroxide and oxygen molecules (Chien and Lin 2019; Pietryczuk et al. 2014; Pokora et al. 2003; Wang and Ki 2020). Catalase (CAT) and peroxidase (POD) enzymes take the next step to convert this active component (peroxide) into harmless water molecules. They reduce two molecules of hydrogen peroxide into two molecules of water and one molecule of oxygen. However, POD could decompose other peroxide compounds (e.g. organic hydroperoxides), via several substrates (e.g. pyrogallol, ascorbate, etc.) as an electron donor, to less harmful compounds. Both CAT and POD were stimulated under stress conditions to mitigate the harmful effect of increased ROS and protect the cells against oxidative damage (Cheng et al. 2016; Hu et al. 2012; Movafeghi et al. 2019; Torres et al. 2008).

According to their metal cofactor, SODs are divided into three basic, widely distributed, groups: iron (FeSOD), manganese (MnSOD), and copper-zinc (Cu/ZnSOD). A fourth isoform, NiSOD, was discovered in *Streptomyces* and cyanobacteria (Hu et al. 2012; Pokora et al. 2003; Wang and Ki 2020). In plants, different isoforms of SOD (encoded by multiple genes) were identified in several subcellular locations of the same species. For instance, Arabidopsis has eight known SODs: two MnSODs (MSD1-2), three Cu/ZnSODs (CSD1-3), and three FeSODs (FSD1-3). These isoforms were found in the chloroplasts (CSD2 and FSD1-3), the mitochondria (MSD1), the peroxisomes (CSD3), the cytoplasm (CSD1 and FSD1), the nucleus (FSD1), and the apoplast (MSD2) of the plant cells (Chen et al. 2022; Kliebenstein et al. 1998; Lee et al. 2022). Moreover, five SOD isoforms (SOD1-5) were identified in the leaves of the perennial ryegrass (*Lolium perenne*). However, the specificity of these isoforms was not identified (Hu et al. 2012). Regarding the green algae (Chlorophyta), six SOD genes (five encoding MnSODs and one encoding FeSOD) were identified in the genome of the model alga *Chlamydomonas reinhardtii*. However, there is no evidence for any Cu/ZnSOD encoding genes (Page et al. 2012). Meanwhile, one MnSOD, one Cu/ZnSOD, and two different groups of three FeSODs (depending on the strain) were identified in *Scenedesmus* species (Pokora et al. 2003).

The increased activity of SOD (in addition to other antioxidants) in different algae of Cyanophyta, Chlorophyta, Rhodophyta, and Bacillariophyta under heavy metal and salinity stress has been reported in the literature. However, high doses of heavy metals or salts and/or prolonged exposure may lower the SOD activity (Antoni et al. 2021; Nowicka 2022; Önem et al. 2018; Pietryczuk et al. 2014; Zhao et al. 2022). Yet, despite the studies that highlight the essential role of FeSOD under different heavy metals and salinity stress (Mallik et al. 2011; Okamoto et al. 2001; Pinto et al. 2003), further investigation is needed to identify, and discriminate between the SOD isoforms present in different eukaryotic algae and to compare their roles under environmental stress.

*Pseudochlorella pringsheimii* (Shihar & Krauss) Darienko & al., a green eukaryotic alga, is common in diverse environments including aquatic or terrestrial habitats. It reproduces expeditiously by autospores and has the ability to synthesize and store important stress metabolites (Darienko et al. 2010; Hirooka et al. 2014; Ismaiel 2016). It was shown to have a high tolerance to nitrogen and pH (Hirooka et al. 2014; Ismaiel 2016), heavy metals (Ismaiel and Said 2018), and osmotic stress, and is therefore regarded as an environmental bioindicator (Darienko et al. 2010). Previously, *P*. *pringsheimii* was shown to have three SOD isoforms viz. Cu/ZnSOD, MnSOD and FeSOD (Ismaiel and Piercey-Normore 2019). In addition, the *FeSOD* was characterized and expressed in an external bacterial host which yielded constant high levels of activity (Ismaiel and Piercey-Normore 2019). However, the variation of the activity levels of these SOD isoforms may have been due to undetected stress and therefore further investigation is needed to compare different types of stress.

Accordingly, the aims of this study were (1) to investigate the activity of SOD isoforms of *P*. *pringsheimii* under iron and salinity stress; (2) to follow the expression level of the *FeSOD* gene under these stressors, and (3) to check the activity of CAT and POD as supportive antioxidant enzymes.

## Materials and methods

### Alga, culture, and stressors

The organism used in this study is the green alga *Pseudochlorella pringsheimii* MIYA 102 (Trebouxiophyceae, Chlorophyta), grown in the Phycology Lab, Faculty of Science, Zagazig University, Egypt. Bold’s Basal culture medium was used to support the growth of the investigated alga, as described previously by Bischoff and Bold (1963).

Aliquots of the culture medium (49 ml) were dispensed in 125-ml size Erlenmeyer flasks. Two stressors were selected; iron (FeSO_4_.7H_2_O) and salinity (NaCl) and applied in a range of 0.025-0.700 mM Fe and 8.5–136.0 mM NaCl. The original medium already has standard ingredients of 0.018 mM Fe and 0.430 mM NaCl. After medium dispensation, the flasks were plugged with cotton and sterilized by autoclaving at 121 °C for 20 min. One ml-sized inoculum (5 × 10^5^ cells ml^− 1^ of algal mid-log stock culture) was used to inoculate the cooled culture flasks. The flasks were incubated at 31 ± 0.5 °C and illuminated with continuous fluorescent light at 85 µmol photons m^− 2^ s^− 1^. The algal cells were dispersed routinely, by handshaking, twice daily. The algal growth was monitored every two days as cell count per ml via haemocytometer. After 10 days, the algal cells were harvested by centrifugation at 6,000 *g* for 10 min at 4 °C, (Sorvall Legend X1R, Thermo Scientific). To remove any possibility of adsorbed metals on the cell surface, the pellets were washed with 10 mM Na_2_-EDTA, and by distilled water, weighed (expressed as fresh weight; FW), frozen in liquid nitrogen, and stored at − 20 ˚C until required.

Chemicals used, unless otherwise stated, were of fine analytical grade, purchased from Sigma-Aldrich, Steinheim, Germany.

### Enzyme extraction

Small volumes (1.5 ml) of enzyme extraction buffer (50 mM phosphate buffer pH 7, 0.5% Triton X-100, 1% polyvinylpyrrolidone (PVP), and 1 mM Na_2_-EDTA) were added to the algal pellets, and homogenized by vortexing with an equal volume of glass beads, at 4 °C. The enzyme extracts were eluted by centrifugation at 10,000 *g* for 10 min at 4 °C. The supernatant was used for the *in gel* analysis of the SOD isoforms, and to measure the activity of the antioxidant enzymes.

The enzyme activities were expressed on a protein-content basis. Therefore, the protein content of the extracts was assayed using a Bio-Rad protein assay kit (BioRad, USA) based on Bradford’s method (Bradford 1976), using bovine serum albumin as a standard.

### Determination of the SOD isoforms by activity-stained native PAGE

The activity of SOD isoforms of *P. pringsheimii* were identified via *in gel* SOD staining activity using native PAGE (i.e. conducted in the absence of SDS and β- mercaptoethanol gel-components) using NBT (Beauchamp and Fridovich 1971). Briefly, 20 µl of enzyme extract samples (equivalent to 50 µg protein extract) were run electrophoretically (at 120 V) in 12% native gel with a 4% stacking gel as described by Laemmli (1970), using a mini gel apparatus (BioRad, USA). After 40 min, the gel was soaked in darkness, firstly in 2.45 mM NBT buffer solution (36 mM K- phosphate buffer pH 7.8) for 20 min, and secondly in a 36 mM K- phosphate buffer (pH 7.8) containing 28 µM TEMED (N, N, N’ N’-tetramethylethylenediamine) and 28 µM riboflavin for 30 min at room temperature. Finally, the gel was put under continuous fluorescent light until the appearance of chromatic zones, representing the SOD activity, on a purple-blue background. The *in gel* activity (band intensity of the SOD isoforms under the different treatments) was quantified by a densitometry analysis (via ImageJ software; http://imagej.nih.gov/ij/). The intensities were expressed in arbitrary units and normalized to the control value (set at 100). The values (of each SOD isoform) are mean of three gel replicates ± SD.

### Superoxide dismutase (SOD) activity

The activity of SOD (tube-test) was measured following the procedure of Beauchamp and Fridovich (1971). An aliquot (50 µl) of the enzyme extract was added to the assay buffer consisting of 50 mM phosphate buffer (pH 7.8), 0.1 mM Na_2_-EDTA, 13 mM methionine, 75 µM nitroblue tetrazolium chloride (NBT), and 2 µM riboflavin (added last). The reaction was initiated by placing the sample tubes under cool white fluorescent light (60 µmol photons m^− 2^ s^− 1^). A negative control (wrapped sample tube), and positive control (no sample extract, i.e. no SOD activity) were put in light parallel with the other samples. The optical absorbencies of all sample tubes were measured at 560 nm after 20 min of irradiance and the rate of NBT reduction was calculated for each sample. The negative control was used to detect any misleading reaction of the assay contents, whereas the positive control was used as a reference to calculate the 50% inhibition of the NBT rate reduction for each sample.

One unit of SOD activity was defined as the amount of enzyme required to cause 50% inhibition of the reduced NBT measured at 560 nm in comparison with the positive control under the assay conditions described. The activity was expressed in U mg protein^− 1^.

To discriminate between the activities of the SOD isoforms, inhibitors such as 5 mM H_2_O_2_ (inactivate both Cu/ZnSOD and FeSOD), or 3 mM KCN (inactivate only Cu/ZnSOD) were separately added to the assay reaction and measured as above (Pokora et al. 2003; Wu and Lee 2008).

### Relative expression of ***FeSOD*** transcript

#### RNA extraction

The total RNA of *P. pringsheimii* was extracted using the TRIzol® method (Invitrogen, Carlsbad, CA). Briefly, the algal cells (equivalent to 100 mg of algal fresh weight) of the selected samples were homogenized with 1 ml TRIzol reagent and an equal volume of glass beads 4 °C, and proceeded following the manufacturer’s protocol. The integrity of the RNA was checked by measurement of optical absorbance ratio (A260/280) (using the NanoDrop 2000c spectrophotometer, Thermo Scientific), and by 1% agarose gel electrophoresis. The intact RNA was treated with DNase I (Invitrogen, CA), and checked by PCR cycle for any possibility of DNA contamination. The valid RNA was transcribed to cDNA using the RevertAidTM H Minus First Strand cDNA synthesis kit (Fermentas, USA). All the previous procedures were done according to the manufacturer’s instructions.

### Quantitative reverse transcription PCR (qRT-PCR) analysis of ***FeSOD***

The assay mixture (20-µl volume) consisted of the different cDNA samples (transcribed from 100 ng total RNA), 1X SYBR Green Master Mix (iQTM SYBR® Green Supermix; Bio-Rad), 300 nmol l^− 1^ of each *FeSOD*-specific primer pair (RTCh-HiF and RTCh-HiF product size 144 bp; Table S1) and 18 S rRNA (product size 127 bp) as a reference gene (Table S1).

The thermal amplification cycle consisted of 95 °C for 3 min followed by 40 cycles of denaturation at 95 °C for 30 s, annealing at 58 °C for 30 s, and extension at 72 °C for 30 s. The amplification reaction was done in an MJ Mini™ thermal cycler and monitored by an MJ Mini-Opticon RT-PCR detector (Bio-Rad). To ensure the quality of the PCR product, a melting curve analysis and a negative control (no cDNA sample) were performed, in addition to agarose-gel visualization. The relative levels of *FeSOD* expression were analyzed with the 2^−ΔΔCT^ method as described by Livak and Schmittgen (2001).

### CAT and POD activities

The method of Kar and Mishra (1976) was used to assay the activities of CAT and POD with minor modifications. The activity of CAT was determined by adding 200 µl of the enzyme extract into an assay mixture containing 300 µM phosphate buffer (pH 6.8), and 100 µM H_2_O_2_. After incubation at 25 °C for five minutes, the reaction was stopped by 2% H_2_SO_4_ (v/v) and the residual H_2_O_2_ was titrated against 0.01 N KMnO_4_ until a faint purple color persisted for at least 15 s. A blank was run at the same time in which the enzyme activity was stopped at “zero” time. One unit of catalase activity was defined as the amount of enzyme that decomposes 1 m mol of H_2_O_2_ per minute.

Regarding the POD activity, it was assayed by adding 200 µl of the enzyme extract in 125 µM phosphate buffer (pH 6.8), 50 µM pyrogallol, and 50 µM H_2_O_2_. The mixture was incubated at 25 °C for 5 min after which the reaction was stopped by adding 0.5 ml of 5% (v/v) H_2_SO_4_. The amount of formed purpurogallin was determined by optical absorbance measurement at 420 nm. One unit of peroxidase activity was defined as the amount of enzyme that produces 1 absorbance change at 420 nm per minute. The activity of CAT and POD was expressed as U mg^− 1^ protein.

### Statistical analysis

The data collected in this study were represented as mean value ± SD (standard deviation) of three replicates. All of the statistical analyses were carried out using SPSS 10.0 software (SPSS, Richmond, VA, USA) as described by Dytham (1999). The validity of the data through normal distribution and equality (homogeneity) of variance was assured via the Kolmogorov–Smirnov and Bartlett’s tests, respectively. One-way ANOVA with Duncan’s multiple range tests were employed for the analysis of variance, and to compare the significance level between values at *P <* 0.05. The correlation between the data was tested using Pearson’s bivariate correlation test (cases number; *n* = 39).

## Results

### Algal growth

**Fig. 1:**
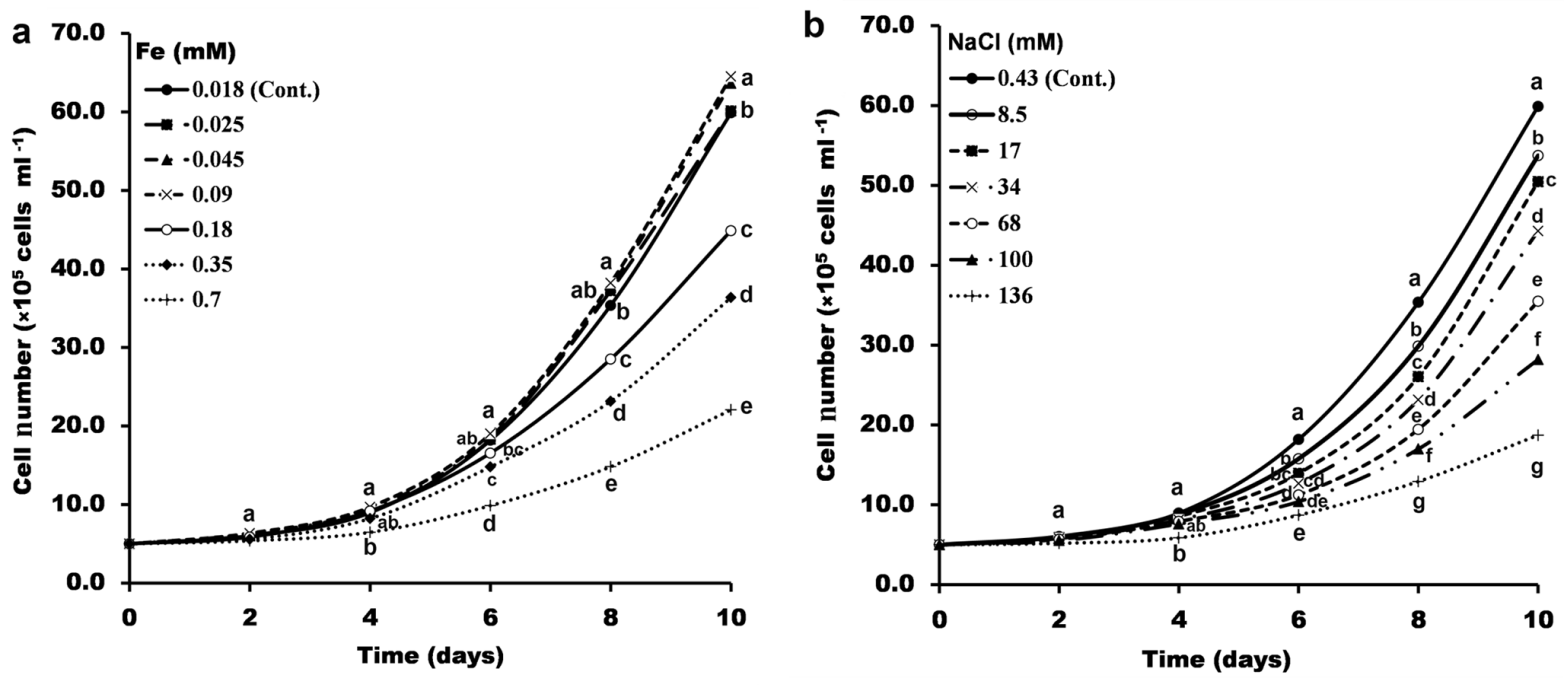
Growth of *P. pringsheimii* (represented by cell number ml^− 1^ culture) under different concentrations of iron (**a**) and NaCl (**b**). Cont.; is the algal growth at the standard medium composition, i.e., 0.018 mM Fe and 0.43 mM NaCl. Each data point represents the mean ± SD of three replicates. The different letters (for each doubling day) represent significant differences at *P* < 0.05 (Duncan’s multiple range test)

The number of cells of *P. pringsheimii* increased by incubation time until the 10^th^ day. The lower iron concentrations (0.025–0.09 mM) had a moderate stimulatory effect on the number of algal cells compared to the control. However, higher iron concentrations (0.18 up to 0.70 mM) reduced the cell number from day 4 until the end of incubation on day 10 (Fig. 1a). Whereas, the different NaCl concentrations studied (8.50 to 136.0 mM) had an inhibitory effect on the algal cell number, compared to the control (Fig. 1b).

### SOD isoforms and activity in ***P. pringsheimii***

**Fig. 2:**
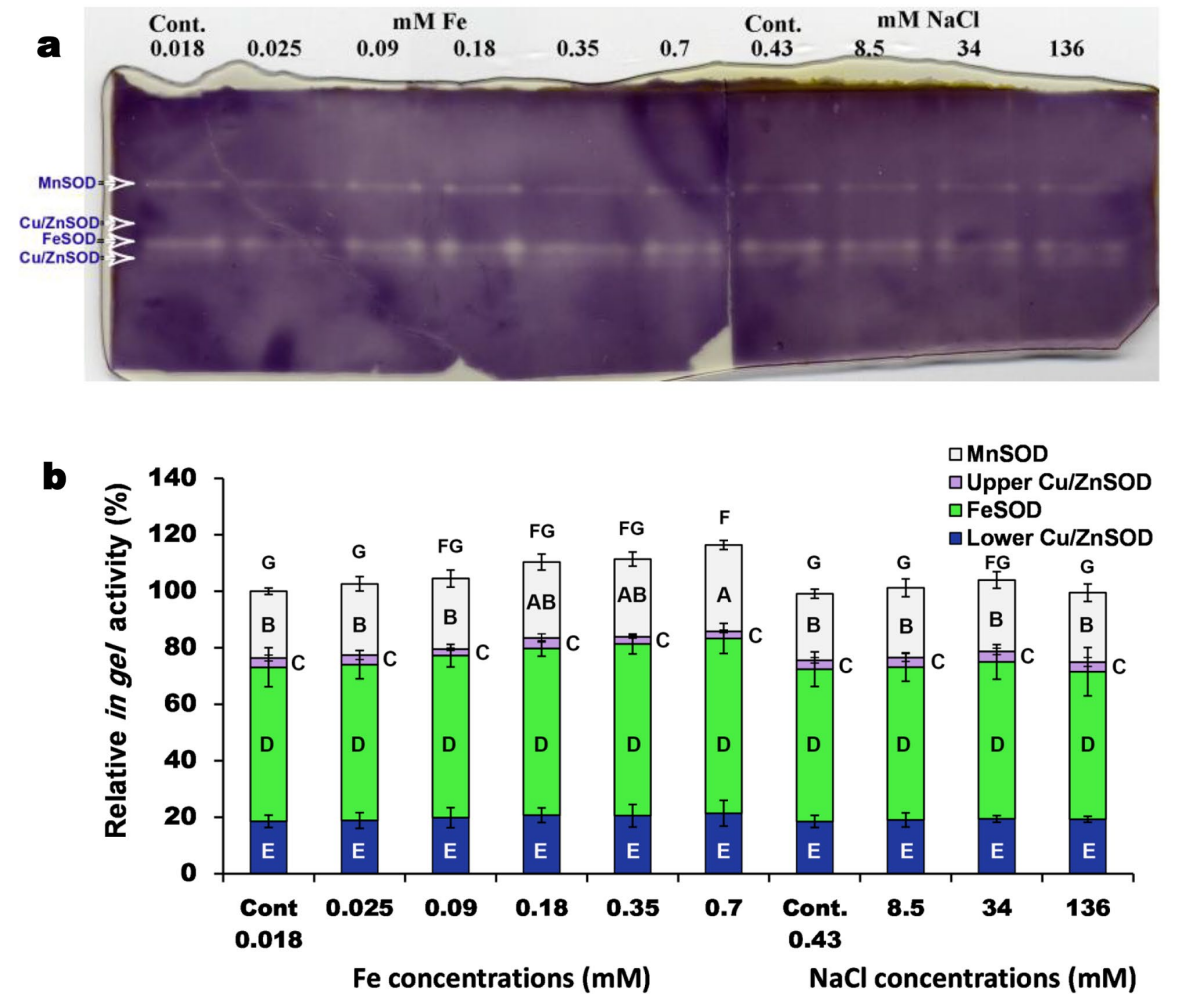
(**a**) Gel activity of the SOD isoforms (native-PAGE gel 12%) of *P. pringsheimii* under different concentrations of iron and NaCl (mM); each lane was loaded with 50 µg protein extract. (**b**) The *in gel* activity (band intensity of the SOD isoforms) quantified by a densitometry analysis (via ImageJ software). The intensities were expressed in arbitrary units and normalized to the control value (set at 100). The values (of each SOD isoform) are mean of three gel replicates ± SD. Cont.; is the algal growth at the standard medium composition, i.e., 0.018 mM Fe and 0.43 mM NaCl. The different letters (for each SOD isoform) represent significant differences at *P* < 0.05 (Duncan’s multiple range test)

The three SOD isoforms (Mn, Fe, and Cu/Zn SOD isoforms) are present in *P. pringsheimii* under the investigated stressors, as suggested by the denaturing gel electrophoresis (Fig. 2). The band intensity of FeSOD, which represents the enzyme activity, was higher than other SOD isoforms. Moreover, the band intensity of all SOD isoforms increased upon the Fe treatment. Whereas those of NaCl treatment was increased until the highest concentration (136 mM NaCl) where a relative reduction (paleness) was observed (Fig. 2, Table S2).

**Fig. 3:**
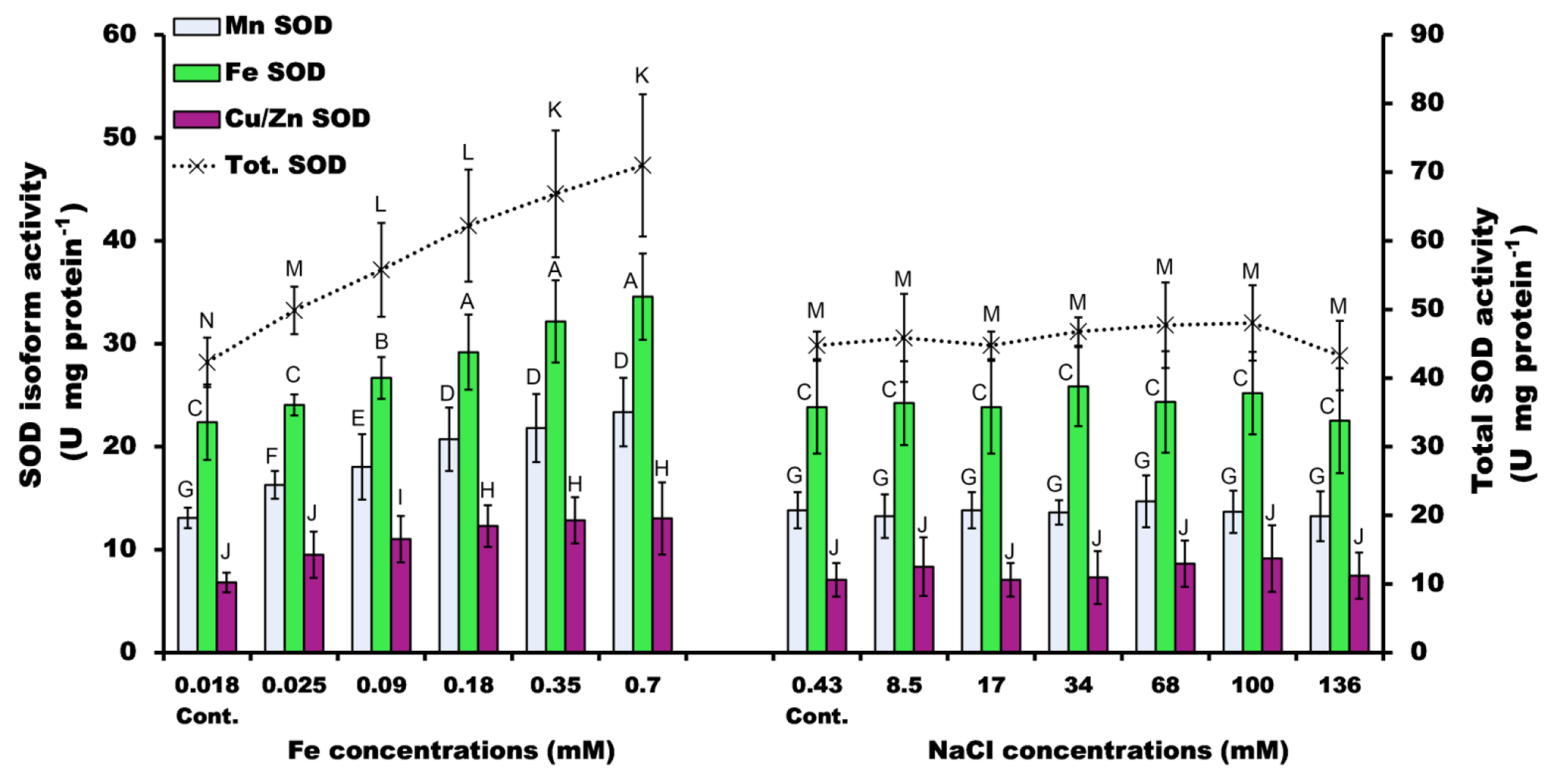
Activity of the different SOD isoforms of *P. pringsheimii* (U mg^− 1^ protein) under different concentrations of iron and NaCl. Cont.; is the algal growth at the standard medium composition, i.e., 0.018 mM Fe and 0.43 mM NaCl. Bars represent the mean ± SD of three replicates. The different letters (for each SOD isoform) represent significant differences at *P* < 0.05 (Duncan’s multiple range test)

The activity of SOD (in total) increased consistently by the different concentrations of iron, but NaCl had very little effect on the activity of SOD with the exception of the highest concentration (136 mM NaCl). The maximum stimulation (71 U mg protein^− 1^) was recorded at 0.7 mM Fe (67.9% above control) (Fig. 3). The activity of the FeSOD isoform was markedly higher than the other two isoforms studied under all concentrations of iron and NaCl, while the activity of MnSOD came second, followed by that of the Cu/ZnSOD isoform (Fig. 3).

### ***FeSOD*** expression increased with high Fe and low NaCl levels

**Fig. 4:**
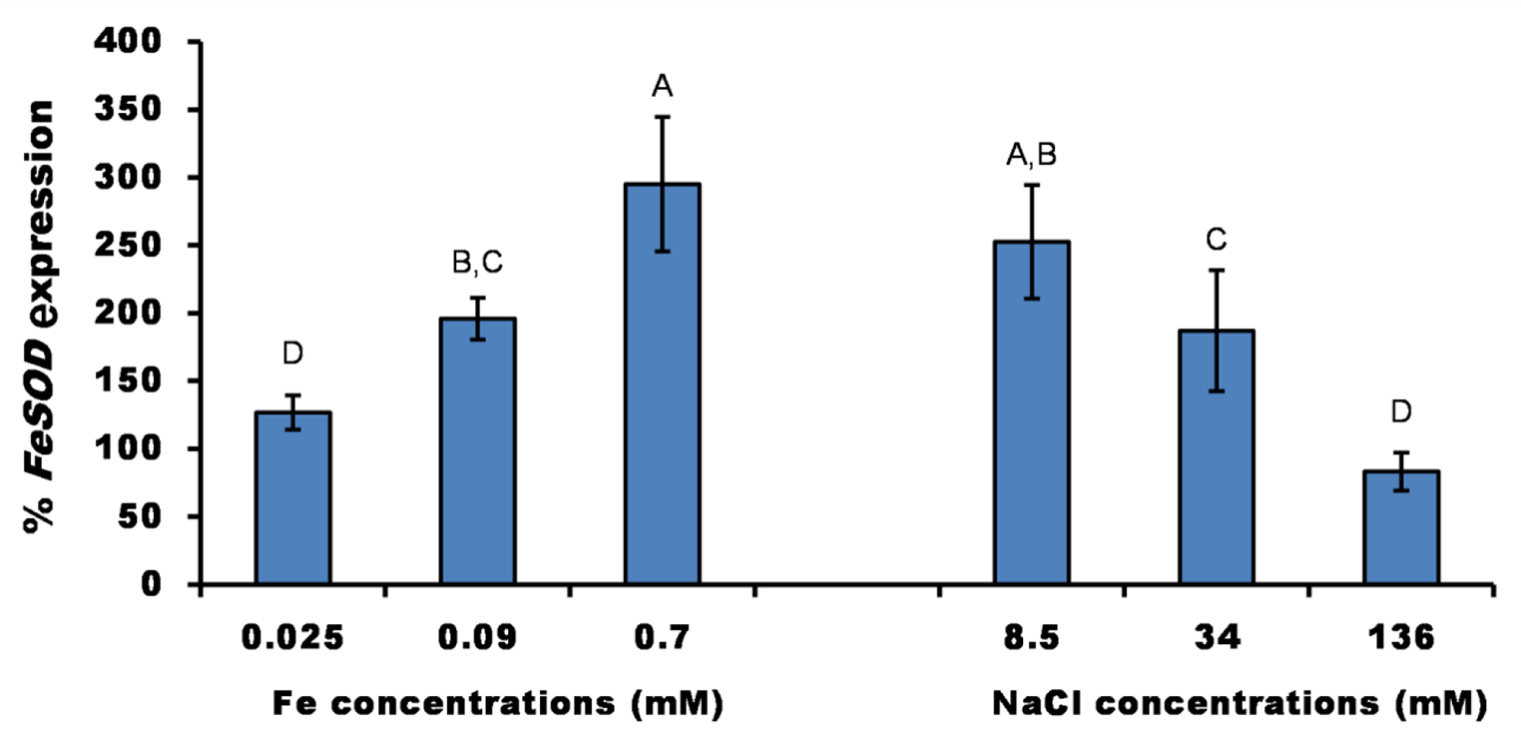
Relative expression of FeSOD transcript of *P. pringsheimii* under different concentrations of iron and salinity. Bars are mean ± SD of three replicates, which were normalized to the control value (the relative expression at standard ion concentrations in the Bold’s Basal medium, 0.018 mM Fe and 0.43 mM NaCl, set at 100). Different letters within each parameter represent significant differences at *P* < 0.05 (Duncan’s)

The relative expression of *FeSOD* showed that high iron concentrations had a stimulatory effect on the expression of the *FeSOD* up to the highest one (3-fold above control). Whereas low NaCl concentrations at 8.5 and 34 mM had a significant (*P* < 0.05) stimulation on the relative expression of *FeSOD*, especially at 8.5 mM NaCl (2.5-fold increase). However, *FeSOD* expression was reduced at the highest NaCl tested concentration (136 mM; Fig. 4).

### CAT and POD activity increased with increasing Fe and NaCl concentrations

**Fig. 5:**
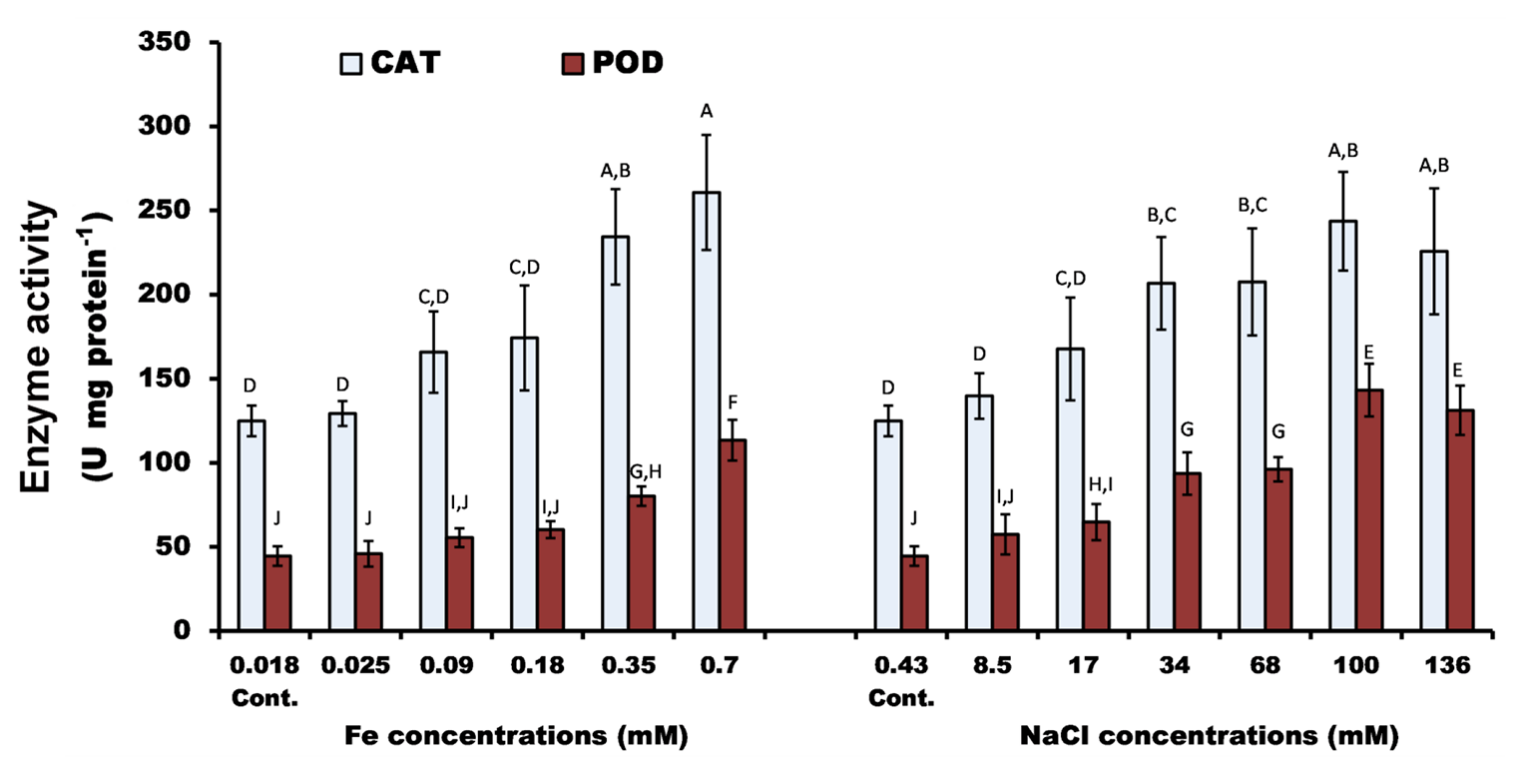
Activity of catalase (CAT) and peroxidase (POD) of *P. pringsheimii* (U mg^− 1^ protein) under different concentrations of iron and NaCl. Cont.; is the algal growth at the standard medium composition, i.e., 0.018 mM Fe and 0.430 mM NaCl. Bars represent the mean ± SD of three replicates. The different letters (for each enzyme) represent significant differences at *P* < 0.05 (Duncan’s multiple range test)

Our data showed that an increase in iron and salinity stress significantly (*P* < 0.05) stimulated the activity of CAT and POD. Where they reached a maximum level at the highest tested concentrations of iron and salinity. However, both enzymes’ activity began to decrease at 136 mM NaCl. The maximum activity values of CAT (2-fold increase above control) and POD (2.5- and 3-fold) were recorded at 0.7 mM Fe, and 100 mM NaCl (Fig. 5).

### Correlation analysis

The results of the correlation between the investigated parameters were represented in Table 1. The high significant positive correlation coefficients (*R*^*2*^ > 0.9; *P* < 0.01) between the activity of total SOD activity and its isoforms activity may indicate the participation of all these isoforms for the total SOD activity against the investigated stressors. In addition, the relative expression of *FeSOD* showed a high significant positive correlation (*R*^*2*^ > 0.66; *P* < 0.01) with the activity of total SOD and its isoforms. However, it showed a low insignificant correlation value between the relative expression of *FeSOD* with CAT and POD activities (*R*^*2*^ = 0.35 and 0.18, respectively). Moreover, a significant positive correlation coefficient (*R*^*2*^ = 0.79; *P* < 0.01) between CAT and POD was also indicated.

**Table 1:** Pearson’s correlation coefficients (*R*^*2*^) between the investigated parameters of *P*. *pringsheimii* under iron and salinity stress

*Parameters*	Cell number	MnSOD	FeSOD	Cu/ZnSOD	Total SOD	*FeSOD* relative expression	CAT
**MnSOD**	-0.122						
**FeSOD**	-0.165	0.907^**^					
**Cu/ZnSOD**	-0.087	0.932^**^	0.911^**^				
**Total SOD**	− 0.183	0.985^**^	0.914^**^	0.931^**^			
***FeSOD*** **relative expression**	− 0.160	0.661^**^	0.734^**^	0.688^**^	0.748^**^		
**CAT**	− 0.820^**^	0.265	0.269	0.197	0.344^*^	0.350	
**POD**	− 0.872^**^	0.008	0.138	0.057	0.084	0.184	0.793^**^

** Correlation is significant at the 0.01 level (2-tailed)

* Correlation is significant at the 0.05 level (2-tailed)

## Discussion

### Algal growth

The induction of the algal cell number by the relatively lower concentrations of Fe may be related to the importance of Fe ions for the algal metabolic activities (as photosynthesis and respiration etc.) and also for the synthesis of significant components such as nucleic acid and proteins (Ismaiel et al. 2018; Nowicka 2022; Sun et al. 2021). Other studies have shown that the exponential growth rate of the green alga *Chlamydomonas reinhardtii* and the diatom *Halamphora luciae* was significantly (*P* < 0.05) reduced only at the higher tested concentration of Cu (100 µM) and Zn (120 µM) (Antoni et al. 2021; Luis et al. 2006), which is consistent with our results. Moreover, Mao et al. (2022) showed that the growth of the green alga *Chlorella thermophila* and the blue-green alga *Leptolyngbya* sp. was negatively affected by increasing As(III) concentrations during 15 days of incubation. The growth of *Leptolyngbya* sp. was lagging on the third day, which was not found in *C*. *thermophila*. Comparatively, in our study, the growth-curve pattern of *P. pringsheimii* produced the largest number of cells at the lowest Fe concentration (Fig. 1a). Regarding the salinity effect, our results were consistent with the literature, where the cell number of *Chlorella vulgaris* was significantly decreased under the increasing concentrations of cadmium-selenide nanoparticles (25 to 50 mg l^− 1^) (Movafeghi et al. 2019) or Cd sulphate salt (0.5-7 mg l^− 1^) (Cheng et al. 2016). The literature also shows an inhibitory dose-dependent effect as a result of increased ROS production, ionic toxicity, and the disruption of osmotic pressure that resulted in nutritional imbalances (Chen et al. 2015; Ismaiel et al. 2018; Mao et al. 2020; Pietryczuk et al. 2014; Sun et al. 2021).

### SOD isoforms

The increased band intensity of FeSOD under stress (Fig. 2) are consistent with the claim that FeSOD was the most important SOD isoform in green algae (Chien and Lin 2019; Luis et al. 2006; Pokora et al. 2003; Wang and Ki 2020). Previously, NaCl induced the appearance of new FeSOD isoforms in *Chlorella* sp. which support its importance, as in our study, against other isoforms such as MnSOD (Mallik et al. 2011). Also, the FeSOD isoform in common bean (*Phaseolus vulgaris*) was induced by salinity, to a higher level over that of MnSOD. Hu et al. (2012) reported the different response of SOD isoforms (Fe-, Mn-, and Cu/ZnSODs) in leaves of the perennial ryegrass (*Lolium perenne*) under the effect of salinity stress. They showed that the majority of SOD isoforms of *L. perenne* had a high activity after the 4th day of exposure, however, the extended exposure had a negative effect on these isoforms.

Our results also showed the presence of the Cu/ZnSOD isoform in *P. pringsheimii* (Fig. 2) which may boost the alga’s ability to withstand stress. This type of SOD isoform was not reported much in eukaryotic algae especially for *Chlorella* species (Asada et al. 1977; Chien and Lin 2019; Mallik et al. 2011; Pelah and Cohen 2005).

### SOD activity

The induction of SOD activity was not surprising under stressors in this study since SOD is known to be an essential enzyme to cope with ROS production. The higher activity of SOD under Fe stress is likely related to the importance of Fe as a co-factor for this enzyme activity (Torres et al. 2008; Wang and Ki 2020). Each of the three separate SOD isoforms showed a similar trend to that described above for total SOD. The activity of Mn, Fe, and Cu/Zn SOD isoforms was increased significantly (*P* < 0.05) by the different concentrations of iron, whereas the NaCl did not significantly (*P* < 0.05) alter the activity of the SOD isoforms compared to the control.

In agreement with the presented observations (Fig. 3), Pinto et al. (2003) reported that heavy metals promote the activity of FeSOD and MnSOD isoforms, but do not appreciably change the activity of the Cu/ZnSOD isoform. This suggests that the Cu/ZnSOD isoform may be triggered by something other than heavy metals, highlighting potentially different roles of these isoforms to protect the electron transport systems in mitochondria and chloroplasts against ROS toxicity. In this regard also, Okamoto et al. (2001) reported the increased activity of the FeSOD of the marine dinoflagellate *Lingulodinium polyedrum* after treatment with metal ions including Cu, Cd, Pb, and Cu. Similarly, exposure of the green algae *C*. *vulgaris, C. reinhardtii*, and *Closterium ehrenbergii* to heavy metals increased the SOD activity (Cheng et al. 2016; Luis et al. 2006; Wang and Ki 2020). In general, the isolated algal strains from metal-contaminated water showed a great rate of SOD activity (Nowicka 2022).

The duration of exposure is another important factor that may affect the survival of algae to stress. The exposure to stress may be divided into two phases; acute (short phase) and chronic (long phase) (Ajitha et al. 2019; Antoni et al. 2021). Usually the metabolic activity of stressed algae increased under the acute phase to resist harsh conditions including high ROS production. However, depending on the tolerance degree of the tested species, the majority of metabolic activity collapsed under the chronic phase of exposure. For example, Zn at 120 µM significantly increased (*P* < 0.05) the activity of SOD in the bacillariophyte *Halamphora luciae* at the 4th day of exposure (acute phase), however prolonged exposure (at the 9th day; stationary or chronic phase) lowered the SOD activity, compared to the control. This may be attributed to the high accumulation (internal concentration) of the metal over time in the algal cells which increases the toxicity and ROS production inside the cells (Antoni et al. 2021). Similarly, the SOD activity in *A*. *platensis*-M2 was increased under mild toxicity of Zn and tin (Sn) metals possibly to deal with the accelerated production of superoxide radicals in the cells (Önem et al. 2018; Torres et al. 2008) suggested that the type of plant or algae, its stage of the life cycle (or the age of the organism), and the sensitivity of the tested enzymes may also explain the disparity in the responses. Our results did not show any response even at the lower end of NaCl concentrations reported in these studies, which may be explained by the species being different. SOD activity by NaCl induction may indicate species-specific and stress-specific responsiveness of SOD activity (Ajitha et al. 2019; Chen et al. 2015; Li et al. 2021; Mao et al. 2020; Nowicka 2022; Önem et al. 2018; Rai et al. 2013).

Pietryczuk et al. (2014) reported the increased SOD activity of *C*. *vulgaris* by relatively low NaCl concentrations (0.1 and 1.0 µM), whereas the higher concentrations (10 and 100 µM NaCl) led to reduced or similar SOD activity as the control. They attributed the change of SOD activity under stress to the species of the investigated algae. In contrast, the activity of the FeSOD isoform was highly stimulated by salinity in the roots and nodules of *Phaseolus vulgaris*, whereas the activity of MnSOD was only slightly induced. However, Cu/ZnSOD activity was slightly stimulated at the level of the nodules and highly reduced in the roots (Jebara et al. 2005). However, prolonged exposure may affect enzyme activity as NaCl was found to be highly toxic to plant cells. For example, the activity of SOD activity in leaves of *Lolium perenne* was increased up to the 4th day of exposure to salt stress (250 mM NaCl); after that, a decline of the activity was observed (Hu et al. 2012). The reduced activity may be related to the toxicity of the surplus ROS concentration, inactivation of the enzyme, or the collapse of the total antioxidant system according to the specific-tolerance of the algal organism (Ajitha et al. 2019; Cheng et al. 2016; Movafeghi et al. 2019).

In summary, the higher induction of SOD under iron rather than salinity stress (Fig. 3) may be explained several ways. First, the importance of iron as a cofactor in the SOD enzyme (FeSOD) supports the increased activity of the enzyme by iron availability (Nowicka 2022). Secondly, the stimulation of ROS produced by the participation of Fe which is catalyzed by the Fenton reaction and Haber–Weiss cycle consequently increased the induction of SOD (Nowicka 2022; Yang et al. 2022). Thirdly, the total capacity of algal antioxidant systems of the investigated alga may be more effective under excess Fe stress rather than NaCl (Ajitha et al. 2019; Ismaiel et al. 2018).

### ***FeSOD*** expression

The results of *FeSOD* expression (Fig. 4) were in accordance with that reported by Wang and Ki (2020); where the relative expression of two genes of *FeSOD* (of the green alga *Closterium ehrenbergii*) was increased by 2-fold (at 0.09 mM) and 1.7-fold (at 0.36 mM) by iron treatment. However, the expression of this gene was reduced (equal to the control) by the highest concentration (0.89 mM Fe). They attributed that to the excess of ROS production by the effect of Fe. In this regard, the transcriptional *FeSOD* and *MnSOD* levels in *C*. *reinhardtii* were increased in a Cu dose-dependent manner at a concentration range of 10–50 µM. However, higher concentrations (up to 200 µM Cu) decreased the relative expression significantly (Luis et al. 2006; Wu and Lee 2008) reported the same results of increased transcript level and activity of *FeSOD* and *MnSOD* of *Ulva fasciata* proportionally by the different concentrations of Cu (0–50 µM). Also, Okamoto et al. (2001) observed an increased level of the FeSOD mRNA of *L. polyedrum* exposed to toxic metals. Furthermore, upon exposure to metals such as Fe, Cu, Mn, and Ni, the SOD-encoding genes were increased in *Closterium ehrenbergii* (Wang and Ki 2020). However, as with the activity of the SOD enzyme, the expression of *FeSOD* depends on the heavy metal type, the concentration of its salt, and time of exposure (Nowicka 2022; Wang and Ki 2020).

In contrast, salinity showed different results in the literature, where the induction of SOD gene expression of the blue-green alga *Microcystis aeruginosa* was reported for low NaCl concentrations of 0-8.5 mM (Chen et al. 2015), consistent with our results. Moreover, Zhuang et al. (2021) reported the increased expression of *CSD2* (a chloroplastic Cu/ZnSOD isoform) and the total SOD activity in the shoots of Arabidopsis under salt stress. This induction was responsible for the reduction of O_2_ֺ^−^ accumulation (which converted to H_2_O_2_) in the plant chloroplast under stress, and thus the stress-response signaling between the chloroplast and the nucleus was continued. Interestingly, they also reported the increased expression of the EGY3 (Ethylene-dependent Gravitropism-deficient & Yellow-green) protein under salt stress which promotes salt-tolerance of the plant by protecting the CSD2 from stress-induced proteolysis.

Reduced *FeSOD* expression by high NaCl concentration (Fig. 4) may be related to the toxic effect of salinity on cellular contents, cellular viability, and hence the process to express *FeSOD* transcripts (Hu et al. 2012; Luis et al. 2006). Accordingly, Hu et al. (2012) disclosed the inhibition of the *FeSOD* transcript in *Lolium perenne* plants by salinity stress particularly under a long duration of exposure. The contradiction between the gene expression rate (Fig. 4) and enzyme activity (Fig. 3) under salinity stress may indicate that the changes in the enzyme activity did not depend only on mRNA expression levels but may be regulated at the posttranscriptional level, which in part results in enzyme inactivation or degradation induced by the effect of stress on the protein stability and modification (Glanemann et al. 2003; Xu et al. 2011). In this context, the increased expression levels of cytosolic *Cu/ZnSOD*, Chl *Cu/ZnSOD*, and *APX* did not agree with the changes (reduction) of enzyme activities of two cultivars of the Kentucky Bluegrass plant under drought stress (Xu et al. 2011).

### CAT and POD activity

The role of CAT and POD is to catalyze the peroxide decomposition to H_2_O and molecular oxygen (Cheng et al. 2016; Movafeghi et al. 2019).

Previous investigations reported the induction of SOD, CAT, and POD in response to heavy metals, which was suggested to be a crucial protective mechanism to reduce cellular oxidative damage in metal-contaminated environments for the green microalga *C*. *vulgaris* (Ajitha et al. 2019; Cheng et al. 2016; Movafeghi et al. 2019; Pinto et al. 2003; Rai et al. 2013). Metals such as Cd-Se nanoparticles (CdSe NPs) were found to increase the activity of CAT and POD of *C. vulgaris* to diminish the effect of toxic peroxide. However, at high concentrations (25–50 mg l^− 1^), the activity may be reduced either due to the direct effect of the metal (denaturing the enzyme structure or decreasing its cellular synthesis) as a result of elevated levels of hydrogen peroxide and superoxide anion (Cheng et al. 2016; Movafeghi et al. 2019). Also, CAT and ascorbate peroxidase (APX) activities of *Ulva fasciata* were increased by a Cu concentration range of 0–20 µM; after which activities leveled out at 50 µM Cu. This induction of enzyme activities was suggested to save the consistency balance of cellular H_2_O_2_ at such higher metal concentrations (Wu and Lee 2008). Moreover, the antioxidant enzyme activities (SOD, CAT) of *C. thermophila* and *Leptolyngbya* sp. were increased by the effect of arsenic metal, but longer exposure to the metal reduced the activity of the antioxidant enzymes. Although the suppression level was not the same for the two species, this highlights variations in their stress-adaptation systems (Mao et al. 2022). Additionally, the strains of the brown seaweed *Ectocarpus siliculosus* isolated from Cu-contaminated sites (EcREP10-11 and Es524) showed high CAT activity in response to Cu treatment than the other algal strain (LIA08-4) of the same species (Sáez et al. 2015).

A rising trend in catalase activity of *C*. *vulgaris* was also seen in parallel to a rise in the medium’s Cr concentration (0-100 µg ml^− 1^), indicating that the H_2_O_2_ degradation process was in operation (Rai et al. 2013). However, CAT activity of *C*. *reinhardtii* showed a slight (non-significant) change in the range of 10 to 150 µM Cu, nonetheless, the higher concentration (200 µM) showed a significant inhibitory effect (Luis et al. 2006).

According to these observations, we could conclude that the response of CAT and POD activity to metal stress is dependent on the tolerance and age of the algal species, the dose (concentration), and the metal-type which cause the oxidative stress (Torres et al. 2008).

A stimulatory effect of salinity stress was observed by antioxidant enzymes of *P*. *pringsheimii* (Fig. 5), which may potentially be considered an adaptive response against the imposed stress (Cheng et al. 2016; Movafeghi et al. 2019). In this regard, Zhao et al. (2022) reported the upregulation of *CAT*, *APX*, and *POD* genes (and hence activity) in the green microalga *Ankistrodesmus* sp. to cope with the increased ROS levels under salinity stress. Our results are in agreement with those of Singh et al. (2018) who noted the direct proportion of CAT activity of the microalgae, *Chlorococcum humicola* and *C. vulgaris*, by all tested NaCl concentrations (25–1000 mM). Furthermore, the NaCl concentration at 255 mM stimulated the CAT activity of *Chlorella* sp. by 1.3-fold with the exception of 85 and 425 mM, which had a negative effect on the activity of CAT (Mallik et al. 2011).

Despite the stimulation of the POD activity by the salinity in the roots and nodules of *Phaseolus vulgaris*, the CAT activity was severely reduced at the nodules, and not detected in the plant roots (Jebara et al. 2005; Mostafa and Tammam 2012) attributed the high CAT and POD activity of *Azolla caroliniana* to be a simultaneous response to cope with the consequences of the increased production of H_2_O_2_ levels by salinity stress. They also suggested that the high enzyme activity was due to the enhancement of new enzyme synthesis and/or the activation of pre-existing enzymes. However, prolonged exposure to stress may have a negative effect on enzyme activity. For example, CAT and POD activities of the leaves of *Lolium perenne* were higher than control plants in response to short-term salt treatment (4 days). In contrast, these activities were decreased by longer exposure to stress (Hu et al. 2012). In this work, the deceleration of CAT and POD activities at the highest tested NaCl concentration (136 mM; Fig. 5) may be due to reaching the critical level of increased oxidative stress imposed on the algal cells (Hu et al. 2012; Wang and Ki 2020).

### Correlation analysis

The correlation of the SOD activities with the expression of *FeSOD* implies that higher gene expression is a factor in increased SOD activity and hence tolerance to the investigated stressors (Wu and Lee 2008). However, the low correlation between the relative expression of *FeSOD* with CAT and POD activities may indicate the relative independence of these parameters to cope with the harmful effect of stress (Hu et al. 2012; Mao et al. 2022). Similarly, the lower values of the correlation coefficients between the algal cell number and the investigated antioxidant parameters may indicate the importance and coherence of these parameters regardless of the stress effect (magnitude) on the growth of the algal cells (Rai et al. 2013). Finally, the significant positive correlation coefficients between CAT and POD may reveal the importance and compatibility of these enzymes to break down the level of toxic hydrogen peroxide and protect algal cells (Ismaiel et al. 2014).

In conclusion, to our knowledge, this is the first study to investigate the SOD isoforms activity of the stress-tolerant green alga *P. pringsheimii* by two methods (*in gel* and in vitro assay) under stress conditions. The three SOD isoforms were induced by iron treatment; while NaCl showed a low non-significant stimulation. Moreover, the activity of FeSOD was higher than that of the other SOD isoforms, and its transcript was highly induced under the tested stressors. The data indicated the potent response of the FeSOD to oxidative stress caused by Fe and NaCl, which is essential for the survival of photosynthetic organisms. The important role of CAT and POD enzymes was also marked under stress conditions. This emphasizes their application as potential molecular biomarkers for toxicity evaluations. Further work regarding non-enzymatic antioxidants, biochemical, and proteomic analyses are currently underway by the authors, in order to provide additional information for the complex response of algae to iron and salinity stressors.

## Electronic supplementary material

Below is the link to the electronic supplementary material.

Supplementary Material 1

## References

[CR1] Ajitha V, Sreevidya CP, Kim JH, Singh IB, Mohandas A, Lee JS, Puthumana J (2019) Effect of metals of treated electroplating industrial effluents on antioxidant defense system in the microalga *Chlorella vulgaris*. Aquatic Toxicol 217:105317 10.1016/j.aquatox.2019.105317 31670168

[CR2] Antoni JS, Daglio Y, Areco MM, Rodríguez MC (2021). Zinc-induced stress on cells of Halamphora luciae (Bacillariophyceae). Eur J Phycol.

[CR3] Asada K, Kanematsu S, Uchida K (1977). Superoxide dismutases in photosynthetic organisms: absence of the cuprozinc enzyme in eukaryotic algae. Arch Biochem Biophys.

[CR4] Beauchamp C, Fridovich I (1971). Superoxide dismutase: improved assays and an assay applicable to acrylamide gels. Anal Biochem.

[CR5] Bischoff HW, Bold HC (1963). Phycological studies. IV. Some soil algae from Erchanted Rock and related algal species. Univ Tex Publication No.

[CR6] Bradford MM (1976). A rapid and sensitive method for the quantitation of microgram quantities of protein utilizing the principle of protein-dye binding. Anal Biochem.

[CR7] Chen L, Mao F, Kirumba GC, Jiang C, Manefield M, He Y (2015). Changes in metabolites, antioxidant system, and gene expression in *Microcystis aeruginosa* under sodium chloride stress. Ecotoxicol Environ Saf.

[CR8] Chen H, Lee J, Lee JM, Han M, Emonet A, Lee J, Jia X, Lee Y (2022). MSD2, an apoplastic Mn-SOD, contributes to root skotomorphogenic growth by modulating ROS distribution in Arabidopsis. Plant Sci.

[CR9] Cheng J, Qiu H, Chang Z, Jiang Z, Yin W (2016). The effect of cadmium on the growth and antioxidant response for freshwater algae Chlorella vulgaris. SpringerPlus.

[CR10] Chien L, Lin W (2019) Changes in photochemical efficiency and differential induction of superoxide dismutase in response to combined stresses of chilling temperature and relatively high irradiation in two *Chlorella* strains. In: Milada Vítová (ed) Microalgae - from physiology to application. IntechOpen. 10.5772/intechopen.89024

[CR11] Darienko T, Gustavs L, Mudimu O, Menendez CR, Schumann R, Karsten U, Friedl T, Pröschold T (2010) *Chloroidium*, a common terrestrial coccoid green alga previously assigned to *Chlorella* (Trebouxiophyceae, Chlorophyta). Eur J Phycol 45:79–95

[CR12] Dytham C (1999). Choosing and using statistics: a biologist’s guide.

[CR13] El-Sheekh MM, Salman JM, Grmasha RA, Abdul-Adel E, Saleh MM, Al-sareji OJ (2022) Influence of Fe^+ 2^ on the biomass, pigments, and essential fatty acids of *Arthrospira platensis*. Biomass Conv Bioref 1–9. 10.1007/s13399-022-02470-9

[CR14] Glanemann C, Loos A, Gorret N, Willis LB, O’brien XM, Lessard PA, Sinskey AJ (2003). Disparity between changes in mRNA abundance and enzyme activity in *Corynebacterium glutamicum*: implications for DNA microarray analysis. App Microbiol Biotechnol.

[CR15] Hirooka S, Higuchi S, Uzuka A, Nozaki H, Miyagishima SY (2014). Acidophilic green alga *pseudochlorella* sp. YKT1 accumulates high amount of lipid droplets under a nitrogen-depleted condition at a low-pH. PLoS ONE.

[CR16] Hu L, Li H, Pang H, Fu J (2012). Responses of antioxidant gene, protein and enzymes to salinity stress in two genotypes of perennial ryegrass (*Lolium perenne*) differing in salt tolerance. J Plant Physiol.

[CR17] Ismaiel MMS (2016). Effect of nitrogen regime on antioxidant parameters of selected prokaryotic and eukaryotic microalgal species. Acta Physiol Plant.

[CR19] Ismaiel MMS, Piercey-Normore MD (2019). Molecular characterization and expression analysis of iron superoxide dismutase gene from Pseudochlorella pringsheimii (Trebouxiophyceae, Chlorophyta). Physiol Mol Biol Plants.

[CR18] Ismaiel MMS, Said AA (2018). Tolerance of *Pseudochlorella pringsheimii* to cd and pb stress: role of antioxidants and biochemical contents in metal detoxification. Ecotoxicol Environ Saf.

[CR20] Ismaiel MMS, El-Ayouty YM, Piercey-Normore MD (2014). Antioxidants characterization in selected cyanobacteria. Ann Microbiol.

[CR21] Ismaiel MMS, Piercey-Normore MD, Rampitsch C (2018). Proteomic analyses of the cyanobacterium *Arthrospira* (*Spirulina*) *platensis* under iron and salinity stress. Environ Exp Bot.

[CR22] Jebara S, Jebara M, Limam F, Aouani M (2005). Changes in ascorbate peroxidase, catalase, guaiacol peroxidase and superoxide dismutase activities in common bean (*Phaseolus vulgaris*) nodules under salt stress. J Plant Physiol.

[CR23] Kar M, Mishra D (1976). Catalase, peroxidase and polyphenol oxidase activities during rice leaf senescence. Plant Physiol.

[CR24] Kliebenstein DJ, Monde RA, Last RL (1998). Superoxide dismutase in Arabidopsis: an eclectic enzyme family with disparate regulation and protein localization. Plant Physiol.

[CR25] Laemmli UK (1970). Cleavage of structural proteins during the assembly of the head of bacteriophage T4. Nature.

[CR26] Lee J, Chen H, Lee G, Emonet A, Kim SG, Shim D, Lee Y (2022). MSD2-mediated ROS metabolism fine‐tunes the timing of floral organ abscission in Arabidopsis. New Phytol.

[CR27] Li J, Li W, Huang X, Ding T (2021). Comparative study on the toxicity and removal of bisphenol S in two typical freshwater algae. Environ Sci Pollut Res.

[CR28] Livak KJ, Schmittgen TD (2001). Analysis of relative gene expression data using real-time quantitative PCR and the 2^–∆∆CT^ method. Methods.

[CR29] Luis P, Behnke K, Toepel J, Wilhelm C (2006). Parallel analysis of transcript levels and physiological key parameters allows the identification of stress phase gene markers in *Chlamydomonas reinhardtii* under copper excess. Plant Cell Environ.

[CR30] Mallik S, Nayak M, Sahu BB, Panigrahi AK, Shaw BP (2011). Response of antioxidant enzymes to high NaCl concentration in different salt-tolerant plants. Biol Plant.

[CR31] Mao X, Zhang Y, Wang X, Liu J (2020). Novel insights into salinity-induced lipogenesis and carotenogenesis in the oleaginous astaxanthin-producing alga *Chromochloris zofingiensis*: a multi-omics study. Biotechnol Biofuels.

[CR32] Mao Q, Xie Z, Irshad S, Zhong Z, Liu T, Pei F, Gao B, Li L (2022) Effect of arsenic accumulation on growth and antioxidant defense system of *Chlorella thermophila* SM01 and *Leptolyngbya* sp. XZMQ. Algal Res 66:102762

[CR33] Mostafa EM, Tammam AA (2012). The oxidative stress caused by NaCl in *Azolla caroliniana* is mitigated by nitrate. J Plant Interact.

[CR34] Movafeghi A, Khataee A, Rezaee A, Kosari-Nasab M, Tarrahi R (2019). Toxicity of cadmium selenide nanoparticles on the green microalga *Chlorella vulgaris*: inducing antioxidative defense response. Environ Sci Pollut Res.

[CR35] Nowicka B (2022). Heavy metal–induced stress in eukaryotic algae—mechanisms of heavy metal toxicity and tolerance with particular emphasis on oxidative stress in exposed cells and the role of antioxidant response. Environ Sci Pollut Res.

[CR36] Okamoto OK, Robertson DL, Fagan TF, Hastings JW, Colepicolo P (2001). Different regulatory mechanisms modulate the expression of a dinoflagellate iron-superoxide dismutase. J Biol Chem.

[CR37] Önem B, Doğru A, Ongun Sevindik T, Tunca H (2018). Preliminary study on the effects of heavy metals on the growth and some antioxidant enzymes in *Arthrospira platensis*-M2 strain. Phycol Res.

[CR38] Page MD, Allen MD, Kropat J, Urzica EI, Karpowicz SJ, Hsieh SI, Loo JA, Merchant SS (2012). Fe sparing and Fe recycling contribute to increased superoxide dismutase capacity in iron-starved *Chlamydomonas reinhardtii*. Plant Cell.

[CR39] Pelah D, Cohen E (2005). Cellular response of *Chlorella zofingiensis* to exogenous selenium. Plant Growth Regul.

[CR40] Pietryczuk A, Biziewska I, Imierska M, Czerpak R (2014). Influence of traumatic acid on growth and metabolism of *Chlorella vulgaris* under conditions of salt stress. Plant Growth Regul.

[CR41] Pinto E, Sigaud-kutner TC, Leitao MA, Okamoto OK, Morse D, Colepicolo P (2003). Heavy metal–induced oxidative stress in algae. J Phycol.

[CR42] Pokora W, Reszka J, Tukaj Z (2003). Activities of superoxide dismutase (SOD) isoforms during growth of *Scenedesmus* (Chlorophyta) species and strains grown in batch-cultures. Acta physiol Plant.

[CR43] Rai UN, Singh NK, Upadhyay AK, Verma S (2013). Chromate tolerance and accumulation in *Chlorella vulgaris* L.: role of antioxidant enzymes and biochemical changes in detoxification of metals. Bioresour Technol.

[CR44] Rybak M, Drzewiecka K, Woźniak M, Ratajczak I, Joniak T (2020). Iron-induced behavioural and biochemical responses of charophytes in consequence of phosphates coagulant addition: threats to lake ecosystems restoration. Chemosphere.

[CR45] Sáez CA, Roncarati F, Moenne A, Moody AJ, Brown MT (2015). Copper-induced intra-specific oxidative damage and antioxidant responses in strains of the brown alga *Ectocarpus siliculosus* with different pollution histories. Aquat Toxicol.

[CR46] Singh R, Upadhyay AK, Chandra P, Singh DP (2018). Sodium chloride incites reactive oxygen species in green algae *Chlorococcum humicola* and *Chlorella vulgaris*: implication on lipid synthesis, mineral nutrients and antioxidant system. Bioresour Technol.

[CR47] Sun Y, Wang S, Liu X, He Y, Wu H, Xie W, Li N, Hou W, Dong H (2021). Iron availability is a key factor for freshwater cyanobacterial survival against saline stress. Environ Res.

[CR48] Torres MA, Barros MP, Campos SCG, Pinto E, Rajamani S, Sayre RT, Colepicolo P (2008). Biochemical biomarkers in algae and marine pollution: a review. Ecotoxicol Environ Saf.

[CR49] Ugya AY, Imam TS, Li A, Ma J, Hua X (2020). Antioxidant response mechanism of freshwater microalgae species to reactive oxygen species production: a mini review. Chem Ecol.

[CR50] Wang H, Ki JS (2020). Molecular identification, differential expression and protective roles of iron/manganese superoxide dismutases in the green algae *Closterium ehrenbergii* against metal stress. Eur J Protistol.

[CR51] Wu TM, Lee TM (2008). Regulation of activity and gene expression of antioxidant enzymes in *Ulva fasciata* Delile (Ulvales, Chlorophyta) in response to excess copper. Phycologia.

[CR52] Xu L, Han L, Huang B (2011). Antioxidant enzyme activities and gene expression patterns in leaves of Kentucky bluegrass in response to drought and post-drought recovery. J Am Soc Hortic Sci.

[CR53] Yang Y, Fan X, Zhang J, Qiao S, Wang X, Zhang X, Miao L, Hou J (2022). A critical review on the interaction of iron-based nanoparticles with blue-green algae and their metabolites: from mechanisms to applications. Algal Res.

[CR54] Zhao Y, Li Q, Gu D, Yu L, Yu X (2022). The synergistic effects of gamma-aminobutyric acid and salinity during the enhancement of microalgal lipid production in photobioreactors. Energy Convers Manag.

[CR55] Zhuang Y, Wei M, Ling C, Liu Y, Amin AK, Li P, Li P, Hu X, Bao H, Huo H, Smalle J (2021). EGY3 mediates chloroplastic ROS homeostasis and promotes retrograde signaling in response to salt stress in Arabidopsis. Cell Rep.

