# Supplementary material for: Antioxidant enzymes of Pseudochlorella pringsheimii under two stressors: variation of SOD Isoforms activity
Source: J Plant Res. 2023 Jun 13;136(5):755–67. doi: 10.1007/s10265-023-01473-5 (PMC10421774; doi:10.1007/s10265-023-01473-5)
Supplement: Supplementary file 1 — Supplementary Material 1 [file 10265_2023_1473_MOESM1_ESM.pdf]

Supplementary materials

Antioxidant enzymes of *Pseudochlorella pringsheimii* under two stressors: Variation of SOD Isoforms activity

Authors names: **Mostafa M. S. Ismaiel[1*], Michele D. Piercey-Normore[2]**

[1] Botany and Microbiology Department, Faculty of Science, Zagazig University, Zagazig, 44519, Egypt.

[2] Faculty of Science, Algoma University, Sault Ste Marie, Ontario, P6A 2G4, Canada

* Corresponding author; e-mail: mostafamsami@yahoo.com; permanent address: Botany and Microbiology Department, Faculty of Science, Zagazig University, Zagazig, 44519, Egypt.

Tel: +2 0111 7373167

Fax: +2 055 2308213

Table S1. Primers used for the analysis of *FeSOD* transcript of *P. pringsheimii*

Gene	Primers	Sequence	Size (bp)	GenBank accession no.
**Iron superoxide dismutase (*FeSOD*)**	RTCh-HiF	ACCACACTTTCTTCTGGGAGAGCA	144	KX274326
	RTCh-R	AGCCAAACTGAGTGGCACCTG		
**18S rRNA (reference gene)**	18S_ChF	TTGACGGAAGGGCACCA	127	X13688
	18S_ChR	CACCACCCATAGAATCAAGAAAGAG		

Table S2. Relative density (%) of *in gel* SOD isoforms activity of *P. pringsheimii* under iron and salinity stress

Treatment	**Fe (mM)**						**NaCl (mM)**			
SOD Isoform	**Cont. (0.018)**	**0.025**	**0.09**	**0.18**	**0.35**	**0.7**	**Cont. (0.43)**	**8.5**	**34**	**136**
**MnSOD**	23.58^{B}	25.17^{B}	25.02^{B}	26.88^{AB}	27.49^{AB}	30.56^{A}	23.59^{B}	24.67^{B}	25.27^{B}	24.53^{B}
**Upper CuZnSOD Band**	3.31^{C}	3.42^{C}	2.25^{C}	3.71^{C}	2.51^{C}	2.54^{C}	3.14^{C}	3.33^{C}	3.71^{C}	3.45^{C}
**FeSOD**	54.53^{D}	55.19^{D}	57.39^{D}	59.05^{D}	60.81^{D}	61.89^{D}	53.91^{D}	54.16^{D}	55.60^{D}	52.25^{D}
**Lower CuZnSOD Band**	18.58^{E}	18.86^{E}	19.86^{E}	20.73^{E}	20.56^{E}	21.42^{E}	18.53^{E}	19.05^{E}	19.43^{E}	19.29^{E}
**Total SOD**	100.00^{G}	102.65^{G}	104.53 FG	110.37 FG	111.37 FG	116.40^{F}	99.17^{G}	101.22^{G}	104.00^{FG}	99.52^{G}

* The *in gel* activity was quantified by a densitometry analysis via ImageJ software. The intensities was expressed in arbitrary units and normalized to the control value (set at 100). Cont. is the algal growth at the standard medium composition, i.e., 0.018 mM Fe and 0.43 mM NaCl. The values (of each SOD isoform) are mean of three gel replicates ± SD. The different letters (for each SOD isoform) represent significant differences at $P < 0.05$ (Duncan's multiple range test).
